# Assessment of the Antibacterial Effects of Bismuth Nanoparticles against *Enterococcus faecalis*

**DOI:** 10.1155/2020/5465439

**Published:** 2020-10-16

**Authors:** Azita Azad, Sahar Rostamifar, Farzan Modaresi, Ali Bazrafkan, Zahra Rezaie

**Affiliations:** ^1^Oral and Dental Disease Research Center, Department of Oral & Maxillofacial Medicine, School of Dentistry, Shiraz University of Medical Sciences, Shiraz, Iran; ^2^Student Research Committee, School of Dentistry, Shiraz University of Medical Sciences, Shiraz, Iran; ^3^Departments of Microbiology, Advanced Medical Sciences and Technology, And Central Laboratory Research, Jahrom University of Medical Sciences, Jahrom, Iran; ^4^Student Research Committee, Jahrom University of Medical Sciences, Jahrom, Iran

## Abstract

**Introduction:**

*Enterococcus faecalis* (*E. faecalis*) is the most important species in dentistry and plays a significant role in the etiology of persistent apical lesions after root canal treatment. Up to date, the intracanal application of 2% chlorhexidine for 7 days is the best way to eliminate *E. faecalis.* However, due to the ability of this bacterium to persist and survive in harsh environments, many studies have been directed towards finding an alternative strategy for prevention or eradication of it. This study was conducted to investigate the effect of bismuth nanoparticles on *E. faecalis*, as an etiologic factor in recurrent root canal infections.

**Methods:**

Forty patients, referred to Endodontic Ward of Shiraz University of Medical Science for endodontic pretreatment, provided root canal samples. First, all samples were transferred in Enterococcosel broth and incubated. Then, samples which showed growth were plated on blood agar plates and incubated for further PCR procedure. Nanoparticle powder was dissolved in high-purity water, and the final concentration of bismuth nanoparticles (BiNPs) was measured by the spectrophotometer. Minimum inhibitory concentration (MIC) of BiNPs against *E. faecalis* was determined by microbroth dilution method according to methods for antimicrobial susceptibility tests. Also, bactericidal assays were conducted in Mueller-Hinton broth medium and reported as the concentration of BiNPs that reduced the viable bacterial count by 99.9%.

**Results:**

Of all samples, 77.5% revealed the presence of *E. faecalis* by PCR. Also, *E. faecalis* growth inhibition was observed at concentrations ranging from 0.625 *μ*g/ml to 20 *μ*g/ml (geometric mean: 2.337 *μ*g/ml), and the MBC values were between 1.25 *μ*g/ml and 40 *μ*g/ml (geometric mean: 4.781 *μ*g/ml), which in comparison with chlorhexidine, these values were about one-eighth of chlorhexidine.

**Conclusion:**

The experimental data suggest that bismuth nanoparticles could be an interesting alternative to combat *E. faecalis*, which, in view of the advantages mentioned for bismuth nanoparticle like inhibiting *Streptococcus mutans* biofilm formation and higher antibacterial activity compared to chlorhexidine, can be suggested to be used in different fields of dentistry.

## 1. Introduction

*Enterococcus faecalis* as a member of Enterococcus genus is a Gram-positive, facultative anaerobe that most commonly found as the commensal in the gastrointestinal tract (including the oral cavity) and the urogenital system. Of all Enterococci species, *E. faecalis* is the most important species in dentistry due to their role in dental diseases, including endodontic infections, periodontitis, and dental caries [1, 2]. In this regard, studies have shown a significant association of *E. faecalis* with the occurrence of endodontic treatment failures [3]. Unlike primary intraradicular infections, which are polymicrobial and predominated by Gram-negative anaerobic rods, the organisms involved in secondary infections are limited to one or a few bacterial species [4]. *E. faecalis* is a persistent microorganism that, in spite of making a small part of the flora in untreated canals, plays an important role in the etiology of persistent apical lesions after root canal treatment. It is usually found in a high percentage of endodontic failures and it can remain alive in the root canal as a single microorganism or as a main constituent of the flora [4]. The presence of *E. faecalis* is associated with different forms of endodontic infection, such as primary and persistent endodontic infections. In primary infections, it is more often found in asymptomatic chronic periradicular lesions than in acute periradicular periodontitis or abscesses. It is isolated in 4 to 40% of primary endodontic infections, while its frequency in teeth with failed treatment is nine times more [5]. Studies using culture methods for isolation of Enterococci in secondary endodontic infections reported a prevalence between 24 and 77%, whilst using a PCR method results in an isolation rate ranging from 67 to 77% [6], so the molecular methods can be a benefit in this regard due to their high sensitivity and accuracy, among which multiplex polymerase chain reaction (PCR) is one of the latest [7]. Indeed, *E. faecalis* can be one of the substantial factors in root canal treatment failures, and its presence at the time of obturation might significantly reduce the treatment success rates [8]. Its pathogenicity primarily relies upon its survival and persistence in the root canals, although it has additional virulence factors, such as the ability to attach and forms biofilm on host surfaces [9]. Many studies have investigated different endodontic medicaments and irrigant to eradicate and/or prevent *E. faecalis* from gaining access to the root canal system during treatment. For instance, 3% to full-strength sodium hypochlorite can destroy *E. faecalis*, including its existence as a biofilm [10]. EDTA and 10% citric acid solution have a little antibacterial effect against *E. faecalis* activity; however, they have the capability to remove the inorganic part of the smear layer, allowing other irrigants to reach to the dentinal tubules [11]. Also, MTAD, a new root canal irrigant consisting of a mixture of tetracycline, an acid, and detergent, has demonstrated a promising ability to eradicate *E. faecalis* [12]. Likewise, 2% chlorhexidine gel or liquid is effective in eliminating *E. faecalis* from superficial layers and dentinal tubules, which may be assigned to substantive antimicrobial effect [13]. Other irrigants which showed to be effective at eliminating *E. faecalis* include stannous fluoride and ozonated water [14]. On the other hand, calcium hydroxide, a commonly used intracanal medicament, showed to be almost ineffective against these bacteria [15]. Furthermore, the antibacterial activities of several sealers have also been examined against *E. faecalis*, with Roth 811, a zinc oxide eugenol-based sealer, having the strongest inhibitory effects [16]. Until further investigations, the intracanal application of 2% chlorhexidine for 7 days is reported to be the best way to eliminate *E. faecalis* [17, 18]. However, due to the ability of this bacterium to persist and survive in harsh environments as well as the emerging resistance among Enterococcus spices, many studies have been directed towards finding an alternative strategy for prevention or eradication of *E. faecalis* from the root canal system [1]. Amongst these strategies, nanoparticles, typically 0.2–100 nm in size, showed good results as novel antimicrobial agents. Their privilege might be due to their high surface-to-volume ratio, which increases the interactions between these particles and microorganisms, improving their inhibitory effects [19]. Also, the differences in size and surface area between these particles and conventional antimicrobial agents can reduce the chance of developing resistance [19]. Up to now, the metals most frequently used for biomedical applications include gold, titanium, silver, copper, zinc, magnesium, and bismuth [20]. Bismuth (Bi) is a diamagnetic, crystalline, and brittle metal of the VA group, typically found as bismuth sulfide, bismuth oxide, and bismuth carbonate [21]. Studies have shown that bismuth derivatives and its nanoparticulate forms inhibit *Helicobacter pylori* growth by altering their Krebs cycling, amino acid, and nucleotide metabolisms, and they can be used as an antidiarrheal agent to treat nausea, vomiting, and stomach pain [22]. Additionally, it was reported that BiNPs exhibited antibacterial and antifungal activities at concentrations lower than 1 mM and 2 mM, respectively [23, 24]. Also, they can interfere with the biofilm formation of *S. mutans*, the main etiological agent of dental decays [23]. However, bismuth nanoscale particles' potential for application in dentistry has not been extensively studied, and the present study was conducted to investigate the effect of BiNPs on the standard strain and clinical isolates of *E. faecalis*, as an etiologic factor in recurrent root canal infections.

## 2. Methods

### 2.1. Sample Taking

Forty patients, ages from 18 to 45 years old, including 22 males and 18 females, referred to Endodontic Ward of Shiraz University of Medical Science for endodontic pretreatment, provided root canal samples, which were then analyzed for the presence of *E. faecalis*. All samples were obtained from patients who had rooted canal therapy completed more than 1 year ago. Patients who were pregnant, diabetic, smoker, and those requiring pretreatment due to missing canals, broken instruments, perforations, ledges, or calcified root canals were excluded. None of the selected teeth have termini of the root canal filling more than 5 mm short in radiographic findings and periodontal pockets deeper than 4 mm. After supragingival scaling and isolation with a rubber dam, samples were taken by one of the authors as previously described by Gomes et al. [13]. The tooth and the adjacent field were decontaminated with a 2.5% sodium hypochlorite for 30 s each and then inactivated with 5% sodium thiosulfate. As the previous restorations were removed and the access cavities prepared, the pulp chambers were disinfected with 5.25% sodium hypochlorite, and the obturation materials were removed with ProTaper nickel-titanium rotary instruments SX-F2 (WNT, India) under irrigation with sterile saline. The microbial samples were collected by inserting two sterile paper points into the working length of the canal and keeping them in place for 60 s. The debris on the paper points were transferred into sterile 2 ml Eppendorf tubes containing viability medium Gotenberg agar III transport medium and evaluated immediately within 2 hrs. After shaking the samples in a mixer for 60 s (Vortex, Scientific Industries Inc., Springfield, MA), 1 ml of each sample were used for culture, and the other 1 ml were frozen at −20°C for by PCR procedures [13]. Additionally, a standard strain of *E. faecalis* (ATCC 51299) obtained from the American Type Culture Collection was studied.

The study was approved by the Ethics Committee of Shiraz University of Medical Science (96-01-03-14924). Patients were informed of the study procedures and goals, and written consent was obtained.

### 2.2. Culture and Identification Procedures

First, all samples were transferred into Enterococcosel broth (HiMedia, India) and incubated for 72 h at 35°C. Then, samples which showed growth were plated on blood agar plates (Plast Labor, Rio de Janeiro, RJ, Brazil) and incubated at 35°C for further usages [8].

### 2.3. PCR Procedures

One-milliliter aliquots from positive cultures in Enterococcosel broth (HiMedia, India) were transferred to microtubes, and DNA was extracted by boiling as described by Siqueira and Rocas (2004). Gene that encode for 16S rRNA of *E. faecalis* was targeted. The entire PCR products were loaded into 1% agarose (Cinnagen, Tehran, Iran) gel and electrophoresed (Akhtarian, Tehran, Iran) for 1-1.5 h in the 1 × TAE buffer along with a molecular weight marker. After staining with ethidium bromide (Merck), the DNA bands were visualized under UV illumination (UVP Gel Documentation, Upland, CA, USA), and the prevalence of *E. faecalis* was reported as the percentage of cases investigated. Primer sequence and PCR condition used for identification of *E. faecalis* is shown in Table 1 [25].

### 2.4. Preparation and Characterization of BiNP Solution

Eight milligrams of nanoparticle powder (Nano Scientific Co, USA) were dissolved in 200 ml of high-purity water and sonicated (Biometra, Germany) for 20 minutes at 900 watts. The sonicated solution was sterilized by passing through a 0.2 *μ*m filter (Control Biogene, Spain), and the final concentration of BiNPs was measured by the spectrophotometer (Eppendrof, Germany). The shape, size, and distribution of synthesized BiNPs have been characterized by the high-resolution transmission electron microscopy (TEM) with a JEM 1011 microscope (JEM-1011 “JEOL LTD,” Japan).

### 2.5. Bacterial Suspension

Overnight grown cultures of *E. faecalis* strains in TSA broth (HiMedia, India) at 37°C were centrifuged at 6000 rpm for 4 min (Eppendrof, Germany), and the supernatants were discarded. The collected cells were washed twice with sterile distilled water. Subsequently, the cells were suspended in 5 ml of TSA broth and incubated for 4 h at 37°C, and their concentrations were adjusted to match the turbidity of 0.5 McFarland using a spectrophotometer.

### 2.6. Antimicrobial Susceptibility Testing

Minimum inhibitory concentration (MIC) of BiNPs against *E. faecalis* was determined by microbroth dilution method according to methods for antimicrobial susceptibility tests for bacteria that grow aerobically, 11th edition. Starter cultures of *E. faecalis* were grown at 35°C for 4 h at 200 rpm and used to make 0.5-McFarland standard suspensions, which were further diluted at a ratio of 1 : 100 (5 × 10^5^ CFU/ml) in Mueller-Hinton broth medium (HiMedia, India). In brief, 10 *μ*l of bacterial inoculum was added to Mueller-Hinton broth medium containing 100 *μ*l of serial dilutions of BiNPs in the wells of microtiter plates (Cinnagen, Tehran, Iran). The final concentration of BiNPs ranged from 40 to 1 *μ*g/ml. One hundred microliters of culture medium with 10 *μ*l of the bacterial inoculum without BiNPs was used as a negative control group. Also, chlorhexidine 2% (Oral-B, USA) ranging from 100 to 1 *μ*l/ml was used as the positive control. The plates were incubated at 37°C for 24 h, and the minimum inhibitory concentrations were visually determined and represented as the lowest concentration of the BiNPs that inhibited the bacterial growth as compared with control groups. Each experiment was performed in triplicate. Also, bactericidal assays were conducted in a Mueller-Hinton broth medium. Minimal bactericidal concentrations were reported as the concentration of BiNPs that reduced the viable bacterial count by 99.9% at 24 h of incubation. Viable bacterial counts were determined by standard plating on Mueller-Hinton agar media [26].

## 3. Results

### 3.1. Identification of *E. faecalis* by PCR Procedure

**Figure 1:**
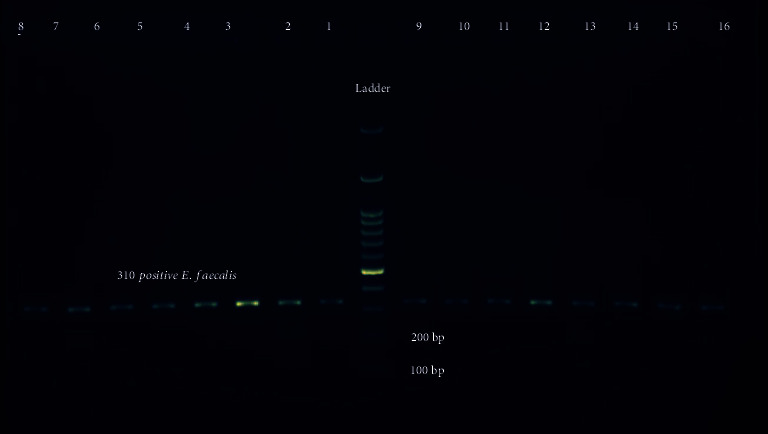
Electrophoresis of *E. faecalis* 16S rRNA gene on 1% agarose gel. The target gene was of 310 bp. Lane 1: control positive. Lanes 2–16: PCR product of 16S rRNA gene (310 bp); ladder: 100 bp DNA size marker.

Forty adult patients consisted of 22 men and 18 women with a mean age of 30.175 (range from 18 to 45 years) were provided samples in the study. Thirty-one out of 40 samples (77.5%) revealed the presence of *E. faecalis* by PCR identification technique loaded into 1% agarose electrophoresis gel (Figure 1). However, eleven root canal samples (22.5%) showed no growth of bacteria. Details regarding subjects were presented in Table 2.

### 3.2. BiNPs Characterization

**Figure 2:**
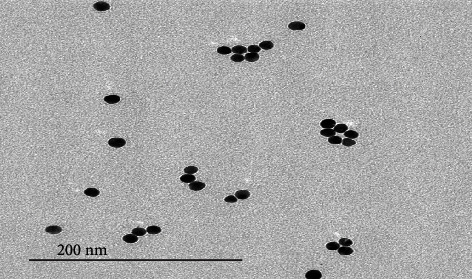
High-resolution transmission electron microscopic image of an isolated bismuth nanoparticles performed by JEOL Jem 1011 Electron Microscope.

Water dispersion of BiNPs has been produced with the aim of their antimicrobial activity estimation against *E. faecalis*. By transmission electron microscopy, it has been found that nanoparticles have an average particles' size of 40 nm with spherical form (Figure 2).

### 3.3. Antimicrobial Susceptibility Testing

To explore the possible antibacterial activity of bismuth nanoparticles, their effect on *E. faecalis* growth was determined. The results showed *E. faecalis* growth inhibition at concentrations ranging from 0.625 *μ*g/ml to 20 *μ*g/ml (geometric mean: 2.337 *μ*g/ml). Also, the MBC values were between 1.25 *μ*g/ml and 40 *μ*g/ml (geometric mean: 4.781 *μ*g/ml). Regarding the standard strain of *E. faecalis*, the MIC and MBC values of BiNPs were 5 *μ*g/ml and 10 *μ*g/ml, respectively. This was while the MIC and MBC values of chlorhexidine were recorded as 40 *μ*l/ml and 80 *μ*l/ml.  Table 3 shows the MIC and MBC value of all tested isolates towards BiNPs.

## 4. Discussion

Amongst the Enterococci species isolated from root canals, *E. faecalis* is the most common species; however, it constitutes a small proportion of the microbial species isolated from root canals. In this study, the prevalence of *E. faecalis* among the selected patients was recorded as 77.5%. Also, previous researches studying the existence of *E. faecalis* in root-filled teeth with periapical lesions have reported a wide range of prevalence from 24 to 77% which can be due to the differences in the methods of identification [5, 27, 28]. Additionally, other studies have isolated *E. faecalis* in teeth with prior unsuccessful treatment with a range of 30% to 90% [5, 29]. Most of these studies have been conducted using culturing methods. These methods have provided great insight into the microbiology of endodontic diseases. However, molecular studies have several superiorities when compared with culture. Molecular methods, particularly, polymerase chain reaction (PCR), are more accurate, more predictable, more sensitive, and faster techniques, which may justify the higher prevalence of *E. faecalis* in this study [2]. Antimicrobial drug resistance among Enterococci species has encouraged development of alternative therapeutic strategies. Amongst these strategies, nanomaterials have turned up as noteworthy and innovative antimicrobial agents [30]. Transmission electron microscopy (TEM), high-resolution TEM (HRTEM), and low-resolution TEM (LRTEM) had facilitated the characterization of NPs and revolutionized their use [31]. Due to the advantages of nanoparticles, several studies have been conducted on the antibacterial effects of nanoparticles, most of which on silver nanoparticles. For example, the study of Sadeghi et al. regarding the comparison of the antimicrobial effect of silver nanoparticles and chlorhexidine on *Streptococcus sanguis* and *Actinomyces viscosis* showed that silver nanoparticle had an antimicrobial effect better than chlorhexidine [32]. Furthermore, in the study of Niakan et al., they compared the effect of silver nanoparticles with Deconex disinfectants on *Staphylococcus aureus* and *Pseudomonas aeruginosa* and found that silver nanoparticles antimicrobial effect is superior [33]. In this work, we focused on the effectiveness of bismuth nanoparticles in inhibiting the growth of *E. faecalis*, by broth microdilution method, a standard method which is more accurate, reliable, and easier to interpret compared to other methods such as well diffusion method [34], although the inhibitory effect of elemental bismuth is detected at comparatively high concentrations because of its limited water solubility. However, lower concentrations can be attained with chelating agents such as dimercaptopropanol. Bismuth-dimercaptopropanol has high solubility with reduced antimicrobial activity [35]. Nevertheless, our results showed more effective antibacterial activity of BiNPs against *E. faecalis* in comparison with chlorhexidine, in a way that the MIC and MBC of bismuth nanoparticles were about one-eighth of chlorhexidine.

Chlorhexidine, as a gold standard for the removal of microbial plaque, has a strong antiseptic effect, and studies comparing the effect of chlorhexidine with other antibacterial products show that chlorhexidine has a stronger antibacterial effect on dental plaque microorganisms than other substances. For example, Haffajee et al. showed that chlorhexidine mouthwash has a stronger antimicrobial effect than herbal mouthwashes [36]. However, it can cause complications such as discoloration of the teeth and tongue, taste disturbance, and it also increases the risk of oropharyngeal cancers due to the high alcohol content (12%), which necessitates the use of other therapeutic strategies [37]. Overall, the experimental data suggest that bismuth nanoparticles could be an interesting alternative to combat *E. faecalis*, which, in view of the advantages mentioned for bismuth nanoparticle like inhibiting *Streptococcus mutans* biofilm formation and higher antibacterial activity compared to chlorhexidine, can be suggested to be used in different fields of dentistry. However, there is still insufficient research into long-term effects as well as its complications on human beings, and its use requires more extensive studies. In addition, the compatibility of nanoparticles with the nature and the method of their recovery requires more researches.

## 5. Conclusion

The experimental data suggest that bismuth nanoparticles could be an interesting alternative to combat *E. faecalis*, which, in view of the advantages mentioned for bismuth nanoparticle like inhibiting *Streptococcus mutans* biofilm formation and higher antibacterial activity compared to chlorhexidine, can be suggested to be used in different fields of dentistry.

## Figures and Tables

**Table 1:** Oligonucleotide used in this study for identification of *Enterococcus faecalis* by PCR [25].

Target DNA	Sequence of primer (5′-3′)	Condition	Amplicon size (pb)
16S rRNA	GTTTATGCCGCATGGCATAAGA G CCGTCAGGGGACGTTCAG	95°C -2 min; 36 cycles (95°C -30 s; 60°C -60 s; 72°C 60 s) and 72°C -2 min	310

**Table 2:** Characteristics of the patients and presence of *E. faecalis*.

Patient no.	Age (year)	Gender (M/F)	Presence of *E. faecalis*	Patient no.	Age (year)	Gender (M/F)	Presence of *E. faecalis*
1	19	M	+	21	43	F	+
2	29	F	+	22	31	M	+
3	20	M	+	23	30	M	+
4	41	M	-	24	28	F	-
5	22	F	+	25	20	F	+
6	18	F	+	26	45	M	+
7	45	F	-	27	45	M	+
8	40	M	+	28	33	F	-
9	33	M	+	29	20	M	+
10	30	M	+	30	29	M	+
11	32	M	+	31	30	F	+
12	39	M	+	32	36	F	+
13	19	F	-	33	37	F	+
14	27	M	-	34	20	M	+
15	21	F	+	35	19	M	-
16	35	M	+	36	24	F	-
17	19	F	+	37	34	M	+
18	37	F	+	38	31	M	+
19	26	M	-	39	24	F	+
20	36	M	+	40	40	F	+

**Table 3:** MIC and MBC values of BiNPs against *E. faecalis*.

Patient no.	Presence of *E. faecalis*	The MIC of BiNP (*μ*g/ml)	MBC of BiNP	Patient no.	Presence of *E. faecalis*	The MIC of BiNP (*μ*g/ml)	MBC of BiNP
1	+	2.5	5	21	+	1.25	2.5
2	+	5	10	22	+	5	10
3	+	1.25	2.5	23	+	5	5
4	-	-	-	24	-	-	-
5	+	2.5	5	25	+	2.5	5
6	+	5	5	26	+	1.25	5
7	-	-	-	27	+	1.25	5
8	+	20	40	28	-	-	-
9	+	2.5	5	29	+	5	10
10	+	1.25	2.5	30	+	2.5	5
11	+	0.625	2.5	31	+	0.625	1.25
12	+	1.25	5	32	+	0.625	1.25
13	-	-	-	33	+	5	10
14	-	-	-	34	+	2.5	5
15	+	5	10	35	-	-	-
16	+	10	20	36	-	-	-
17	+	2.5	5	37	+	1.25	1.25
18	+	0.625	1.25	38	+	0.625	1.25
19	-	-	-	39	+	5	10
20	+	1.25	2.5	40	+	10	20

## Data Availability

The experimental and clinical data used to support the findings of this study are included within the article.
